# Swarm Optimization for Energy-Based Acoustic Source Localization: A Comprehensive Study

**DOI:** 10.3390/s22051894

**Published:** 2022-02-28

**Authors:** João Fé, Sérgio D. Correia, Slavisa Tomic, Marko Beko

**Affiliations:** 1 COPELABS, Universidade Lusófona de Humanidades e Tecnologias, Campo Grande 376, 1749-024 Lisboa, Portugal; 19389@ipportalegre.pt (J.F.); slavisa.tomic@ulusofona.pt (S.T.); 2 VALORIZA—Research Centre for Endogenous Resource Valorization, Instituto Politécnico de Portalegre, Campus Politécnico n.10, 7300-555 Portalegre, Portugal; 3 Instituto de Telecomunicações, Instituto Superior Técnico, Universidade de Lisboa, 1049-001 Lisboa, Portugal; beko.marko@gmail.com

**Keywords:** swarm optimization, acoustic localization, embedded programming, wireless sensor network, metaheuristic, edge computing

## Abstract

In the last decades, several swarm-based optimization algorithms have emerged in the scientific literature, followed by a massive increase in terms of their fields of application. Most of the studies and comparisons are restricted to high-level languages (such as MATLAB^®^) and testing methods on classical benchmark mathematical functions. Specifically, the employment of swarm-based methods for solving energy-based acoustic localization problems is still in its inception and has not yet been extensively studied. As such, the present work marks the first comprehensive study of swarm-based optimization algorithms applied to the energy-based acoustic localization problem. To this end, a total of 10 different algorithms were subjected to an extensive set of simulations with the following aims: (1) to compare the algorithms’ convergence performance and recognize novel, promising methods for solving the problem of interest; (2) to validate the importance (in convergence speed) of an intelligent swarm initialization for any swarm-based algorithm; (3) to analyze the methods’ time efficiency when implemented in low-level languages and when executed on embedded processors. The obtained results disclose the high potential of some of the considered swarm-based optimization algorithms for the problem under study, showing that these methods can accurately locate acoustic sources with low latency and bandwidth requirements, making them highly attractive for edge computing paradigms.

## 1. Introduction

In the last decade, swarm optimization methods have found their way into the scientific community, where several algorithms have been proposed and applied in real-life problems. In computer science, swarm optimization assumes a set of sequential operations, where a candidate population is iteratively improved according to a measure of quality (the cost/objective/fitness function). As the opposite of gradient-based optimization [1], these algorithms assume no knowledge of the problem itself, and a candidate population evolves only according to the evaluation of a given cost function. As such, the method uses a combination of random choices and historical knowledge of past results to guide and drive its evolution through the search space, providing a sufficiently good solution, but without the guarantee of achieving a global solution (metaheuristics). The use of metaheuristics to solve optimization problems goes back to the 1970s with the work of J. Holland and the proposal of genetic algorithms (GAs) [2]. The method consisted of a search heuristic based on Charles Darwin’s theory of natural evolution [3]. The proposed methodology reflected the process of natural selection where the fittest individuals are selected for reproduction to produce the next generation. Although still widely applied nowadays [4], GAs, together with simulated annealing (SA) [5] or hill-climbing methods [6], have shown slow convergence towards sub-optimal solutions [7]. Though GA and SA are related to biological evolution and physical proprieties of materials, respectively, the first algorithm based on swarm intelligence was proposed to mimic the finding of good paths by ants [8]. The search technique was inspired by modeling the swarm intelligence of social ants using a pheromone as a chemical messenger [8]. The *Particle Swarm Optimization* (PSO) algorithm followed [9] with the premise of representing the movement of particles in a bird flock, and it was widely applied and with numerous variants proposed over the years [10]. At the turn of the 21st century, the authors of [11] proposed a music-inspired algorithm called *Harmony Search* (HS), and around 2002, K. M. Passino presented a *Bacteria Foraging* algorithm [12]. In 2004, S. Nakrani and C. Tovey published the *Honey Bee* algorithm [13] that they applied to Internet hosting centers, which was followed by a novel bee algorithm [14] and the *Artificial Bee Colony* (ABC) in 2007 [15]. Later, in 2008, the *Firefly* (FA) algorithm was published, inspired by the flashing behavior of fireflies. In 2009, the *Cuckoo Search* (CS) algorithm [16] was proposed, based on the obligate brood parasitic behavior of some cuckoo species in combination with the Lévy flight behavior of some birds and fruit flies. The decade ended with the publishing of the *Bat* algorithm (BAT), which was inspired by the echolocation behavior of micro-bats [17].

At this stage, the fundamentals of *swarm intelligence* had been established. Simple software agents (or particles) move in the search space of a predetermined optimization problem, where the position of a particle represents a candidate solution to the problem at hand. Each particle evolves by updating its position according to rules inspired by their behavioral models. Those rules rely on the best, current, or past position(s), as well as some randomly generated variables, combined with simple arithmetic. From this point, numerous algorithms have been raised in the scientific literature, and most publishers have created journals dedicated to the subject. As a major feature, the efficiency of a metaheuristic algorithm relies on the right balance between exploration (also known as diversification) and exploitation (or intensification), where exploration describes the ability of the algorithm to leave the current optimum in search of a better candidate, and exploitation is defined as the ability of the algorithm to improve the best solution it has found so far by searching a small area around the current solution [18]. In swarm-based optimization, this balance is archived through the control of a set of parameters with a direct impact on its performance, making them dependent on accurate parameterization [19]. This situation implies that different algorithms may have different performances with regard to the same problem. In addition, since these methods are based on an iterative evolution of the first state of a population, their initialization plays an important role in the performance achieved, and may even imply a lack of convergence [20]. Generally, algorithms are evaluated within a set of mathematical functions considered as representative for a wide range of features, such as convexity, continuity, differentiability, etc., but where the obtained performance cannot be conveyed to real-life physical models. This suggests that choosing a specific algorithm for a certain problem may not be a trivial task, especially due to the large number of swarm-based methods available.

There are many applications in which efficiently (accurately and promptly) solving the localization problem is crucial, such as navigation [21], underwater networks [22], surveillance [23,24], or power systems [25,26]. When considering the fourth industrial transformation and the fundamental advanced digital changes—known as Industry 4.0—robust and precise localization can be seen as a key feature in pervasive systems in future industry and factory applications. More specifically, it is necessary for a wide range of industrial applications to perform the localization of acoustic sources. In addition, sound localization may be a valuable instrument for analyzing the workflow of vital machinery (pumps, motors, electric drives, or fans). Such machinery can be targeted for noise reduction, where its noise footprint can be analyzed and compared between diverse workflows or product life spans [27]. In the context of predictive maintenance, one can find applications for preventing structural failure [28], leak localization [29], or nondestructive localization of cracks [30]. In its early stages, the PSO algorithm [9,10] was also used for solving some acoustic localization problems, namely, those related to the localization of partial discharge sources in power transformers [31,32]. Both non-linear and binary forms of the optimization algorithm were successfully applied [25,33]. The PSO algorithm contributed to the beginning of a new approach to the nonlinear optimization problem. As one of the most cited works in the scientific literature, it is still applied in several fields due to its phase-correcting structure for electromagnetic band-gap resonators [34], time-delay equalizer meta-surface for electromagnetic band-gap resonator antennas [35], or artificial magnetic conductor design [36].

From a physical point of view, several approaches exist for acquiring the necessary signals to achieve the localization of an acoustic source. Solutions based on time of arrival [37], time difference of arrival [38], or direction of arrival [39] are well-known examples in the literature; however, they depend on high-precision hardware for timing purposes or on microphone sensor arrays for angle perception, which might drastically raise the network implementation costs. On the contrary, solutions based on energy measurements are much more flexible to deploy, but are capable of achieving good performance. For this reason, only energy-based localization will be under analysis in the present work (however, the extrapolation to any range-based localization method is straightforward). The energy decay model was initially proposed by conducting field experiments with the sound emitted from an engine [40]. The localization approach considers averaging the energy information of the received acoustic signal data samples, standing out for lower bandwidth, since it is sampled at a much lower rate [41]. The energy-based acoustic location (EBAL) problem has traditionally been approached with deterministic algorithms [42,43]. The least-squares method was applied in [44], considering weighted terms and a correction technique. Although it offers certain gains, principally due to the correction performed, its performance might be severely deteriorated in surroundings with high noise powers because of the neglect of the second-order noise terms. Considering the non-convexity of the EBAL problem, the authors in [45,46] proposed the use of convex optimization methods, namely, by applying semi-definite programming relaxations to convert it into a convex one. By contemplating the solution offered by in [45,46], one understands that it is actually circumvented by resorting to a set of convex relaxations that result in increased computational burden. Therefore, more convenient methods were proposed in [47,48,49], where the authors had to take recourse to second-order cone programming techniques. Even though estimators founded on convex optimization render good performance in general, even in surroundings with large noise powers, their biggest shortcoming was related with their computational burden, which was a polynomial function of the network size. The use of a black-box model, namely, a feed-forward neural network, was proposed in [50], showing equivalent or even improved performance in comparison with state-of-the-art methods while being computational simpler. Nonetheless, these results were obtained in a constrained simulated environment whereby it was possible to generate perfect and abundant training data, something that is not typically available in real scenarios. The use of metaheuristics to tackle the acoustic localization problem, namely, Elephant Herding Optimization (EHO) [51,52], was firstly proposed in [53], and its implementation was validated in [54]. The results in [54] demonstrated that the EHO algorithm could supplant deterministic methods for high values of the measurement noise, as it is computational simpler. Ultimately, taking advantage of specific information about the problem layout to intelligently initialize the population, an improved EHO (iEHO) showed even better accuracy, with good results over a wide range of measurement noises, network size, and even in tracking scenarios [55,56]. This is one of the main reasons for why this work studies swarm-based techniques. Considering (1) the good results obtained by the EHO and iEHO, as well as the vast range of existent swarm-based algorithms, and (2) the performance gain in using an intelligent swarm initialization with EHO, the following questions arise: (1) *Can the performances obtained by the EHO be achieved or even exceeded by other swarm-based algorithms?* (2) *Can the population initialization proposed for the iEHO improve the performance of all swarm-based algorithms?* To answer these questions, a total of 10 swarm-based methods were applied to the EBAL problem and tested in this extensive work.

The biggest advantage of swarm-based methods over deterministic approaches is their low computational cost, making them highly attractive for edge computing paradigms by reducing latency and saving bandwidth. When embedded processing is at stake, either by running the algorithms at the edge of the network or even on the sensors, computational complexity and processing time play an important role in selecting the appropriate method [57]. Since its origin in the late 1990s for delivering video content from edge servers [58], edge computing has shown several advantages concerning the reduction of bandwidth and payload overlay [59]. Referring to the acoustic localization problem, running the location algorithm at the edge of the network allows less traffic (since only calculated coordinates are transmitted) and advantages related to privacy and security (since the architecture provides computing and memory storage options close to the device itself) [60]. Secondly, by allocating all of the processing to the edge, the number of sensor nodes and the covered area can grow without the need for centralized data center processing and networking power to increase. Actually, only the number of edge servers would grow proportionally. Nevertheless, these are much cheaper devices, and because of the distributed computing paradigm, networking congestion that could occur on a centralized data center would be avoided. Finally, to implement the solution on edge devices, it is crucial (and sometimes the only option) to do it using low-level programming languages, since the memory and processing are limited. This further increases the importance of the presented work, where the selected algorithms (with implementations available online, but only in MATLAB^®^ or Python) were implemented from scratch in the C language and tested through exhaustive simulations on several embedded devices. It is common that the localization problem is represented through non-linear, non-differentiable, and non-continuous models, where a metaheuristic supplants its counterparts. Even though these methods recently gained a lot of attention, to the best of our knowledge, no comprehensive study about their effectiveness in tackling target localization exists in the literature. Therefore, this work should be seen as a guide and our initiative to incentivize researchers to tackle the localization problem by applying metaheuristic tools. Hence, this review also adds an important contribution to the current state of the art when it comes to computing the localization problem through swarm-based algorithms on edge platforms.

Based on the above discussion and the results obtained, the main insights and contributions of the present work are summarized as follows: (1) application of several of the most significant and up-to-date swarm-based techniques to the EBAL problem and assessing their performance with regard to convergence and localization error; (2) integration of the intelligent initialization technique proposed in [55] (but only integrated with EHO) with all of these swarm techniques to generally validate the improvements in convergence speed for any swarm algorithm; (3) evaluation of the time efficiency of these methods when executed on embedded processors, thus proving the feasibility of the approach for any real edge computing scenario.

The remainder of the paper is organized as follows. Section 2 defines the methodology adopted for the comprehensive study. Section 3 formulates the theoretical background on both energy-based acoustic localization and swarm-based optimization. Section 4 presents a detailed implementation of the testing procedure with regard to the embedded setup and selected algorithms. Section 5 provides the obtained results and their discussion, and lastly, Section 6 concludes the paper and provides possible future directions of research.

## 2. Methodology

On the one hand, when considering the first steps in swarm-based optimization, algorithms such as *Ant System* [8] and *Particle Swarm Optimization* [9] are immediately noticed. Since they are accepted as the first methods based on swarm intelligence, it is common to reference them as landmarks. Currently, considering the metrics from Google Scholar (https://scholar.google.com/, accessed on 10 September 2020), both exceed several tens of thousands of citations. On the other hand, until the present day, more than two hundred algorithms have been proposed in the literature, which makes the process of choosing an algorithm for a given problem somewhat complex. In order to choose the methods to be implemented in the current study, the databases of several publishers (MDPI, IEEE, Elsevier, Springer, Sage, IOS Press, Science Open, AIP, Inder-Science, Wiley and Sons, Emerald, and Taylor and Francis) were searched to collect the published swarm-based methods, which were ordered by year and number of citations per year while considering their date of publication and Google Scholar for the citation metrics. The landmark PSO and ANT algorithms are usually objects of comparison for new proposals, where novel methods present improvements in relation to the first two; hence, they were not targeted for implementation. Then, *Cuckoo Search* (CS) [16] was considered for implementation, as it was the most cited method published in the first decade of the current century, with a mean value of 464 citations per year. To reduce the time grid in recent years, from this point on, the analysis was performed with a five-year interval. Between 2010 and 2014, three algorithms stood out, namely, the *Grey Wolf Optimizer* (GWO) [61] with 4112 citations (685 citations/year), the *Bat Algorithm* [17] (a total of 3753 citations or 375 citations/year), and the *Teaching–Learning-Based Optimization* (TLBO) algorithm [62] (with a total of 2227 citations or 247 citations/year). As such, the GWO algorithm was considered for implementation as representative of the 2010–2014 time window. Then, for the 2015–2019 quinquennium, the methodology was once again refined, and a year-by-year approach is considered. Based on the metric analysis, in 2015, the *Moth–Flame Optimization* algorithm (MFO) [63] was the most cited method, with a total of 1167 citations or 233 citations/year, and it was selected for implementation. Consequently, the *Whale Optimization* (WOA) [64] and the *Salp Swarm* (SSA) [65] algorithms, with 557 and 298 citations/year in 2016 and 2017, respectively, were considered for implementation. With regard to 2018, two algorithms were considered. Firstly, the *Tree Growth Algorithm* (TGA) [66] with 45 citations or 23 citations/year and, secondly, the *Coyote Optimization Algorithm* (COA) [67] were considered because they were some of the few methods that divide a population into groups, similarly to the *Elephant Herding Optimization* (EHO) [51] algorithm. Similarly and for the same reasons, in 2019, two algorithms were selected for implementation. Firstly, the *Supply–Demand-Based Optimization* (SDO) [68] was chosen for its novelty, and *Enhanced Elephant Herding Optimization* (EEHO) [69] was chosen, as it also considers its population divided and manages it in groups. It should be noted that this latest publication corrected three important flaws (each regarding unjustified convergence towards the search space’s origin, unbalanced exploration/exploitation trade-off, and skewed agent distribution) in relation to its original version, which is why the first EHO [51] method will not be considered. It is worth mentioning that other methods with a higher number of citations were published in 2019, e.g., the *Squirrel Search* [70] or the *Harris Hawks Optimization* [71] algorithms. However, they will not be considered here due to their similarities with other already implemented methods, namely, CS and GWO. Finally, in 2020, several new methods could still be found in various publications. Since considering the number of citations would be highly influenced by the month of publication, this criterion was not taken into account. Instead, the *Momentum Search* Algorithm (MSA) [72] was considered due to its inspiration from a physical principle instead of the behavior of living beings. A complete list of the methods selected for analysis is presented in Table 1, along with the number of citations obtained from Google Scholar (accessed in September 2020). For the citations/year metric, the total number of citations was divided by the publication year and subtracted from the current year. A more detailed list of all of the considered algorithms is presented in Appendix B.

After having stated the criteria for selecting the algorithms to implement, it remains to define the hardware processing platform and the programming language. As previously stated, the present work intends to validate decentralized implementations of the acoustic localization problem (namely, over edge computing), apart from accuracy and feasibility. In other words, the algorithms must run on low-complexity and low-clock-rate processors. As such, the obtained results will be closely in line with practical implementations in real contexts. For that purpose, the completion relies on two main features: (1) all algorithms are implemented in the C language; (2) the code runs on embedded processors. These features contrast with the usual testing procedures developed in high-level languages (most commonly, in MATLAB^®^) and executed on high-performance computers, where issues such as floating points, matrix operations, and mathematical functions (e.g., trigonometric functions) are generally well established. Basically, the fact that good performance and convergence results are obtained on high-level platforms does not guarantee operation on computer systems with lower capabilities, namely, embedded systems. On the contrary, however, validations carried out in an embedded context guarantee the operation of algorithms on high-level platforms, taking into account that the change is towards computational improvement. As such, the present work considers the assessment of the selected swarm-based optimization algorithms on Broadcom^TM^ BCM series processors based on ARM^®^ architectures, which are well known for their use on Raspberry Pi Foundation^TM^ electronic boards.

To comprehensively assess the performance of swarm-based methods applied to the acoustic source localization problem, a wide range of processors with different memory capacities and different clock speeds were considered. The set of hardware modules consisted of several Raspberry Pi modules, which went from 700 MHz to 1.5 GHz clock frequencies, and they had 512 MB to 4 GB of RAM and CPU buses that were 32 and 64 bits wide, running the Raspberry Pi Lite operating system. In total, five different modules were used, and their main features are summarized in Table 2. The applicability of Raspberry Pi modules for edge computing applications has been considered in the literature for smart manufacturing [74], smart agriculture [75], and smart surveillance [76]. Nevertheless, when processing requirements increase, shortcomings in terms of performance have been reported [77]. Thus, the use of more computationally efficient algorithms (with lesser computational requirements) is of major importance.

To conclude, the selected swarm-based methods were evaluated on the five modules, seeking: (1) analysis and comparison of convergence and accuracy; (2) validation of improvements by population initialization; (3) validation and analysis of execution times.

## 3. Theoretical Background

The current section intends to provide the necessary theoretical background on both the formulation of the energy-based localization problem and swarm-based optimization.

### 3.1. Energy-Based Acoustic Source Localization

The energy-based acoustic model, which was initially proposed in [40], implies that the observation at a given sensor *i* decays with a ratio inversely proportional to the distance between the sensor and the acoustic source, according to:

(1) $$y_{i}=\frac{g_{i}P}{||x-s_{i}{\left|\right|}^{\beta }}$$

where $g_{i}$ is the gain of sensor *i*, *P* is the transmitted power, x and $s_{i}$ are the source and sensor coordinates, and, finally, β is a decay propagation factor that is dependent on environmental conditions. For the sake of simplicity, an outdoor scenario without reverberation or reflections is considered here, and thus, β=2 [40]. In the case of a two-dimensional problem, $x=x_{x}{\hat{a}}_{i}+x_{y}{\hat{a}}_{j}$ and $s_{i}=s_{ix}{\hat{a}}_{i}+s_{iy}{\hat{a}}_{j}$, where ${\hat{a}}_{i}$ and ${\hat{a}}_{j}$ are the coordinate unit vectors. The extension to higher-dimensional problems is straightforward. By employing the observations defined in (1), the maximum likelihood (ML) estimator of x can be formulated as [53]:

(2) $$\hat{x}=\underset{\begin{array}{c} x \end{array}}{\mathrm{arg min}}\sum_{i=1}^{N}{\left(y_{i}-\frac{g_{i}P}{||x-s_{i}{\left|\right|}^{2}}\right)}^{2}.$$

ML is one of the most commonly employed estimators [53], since it is asymptotically efficient (for large enough data records). This estimator, however, depends on the noise statistics and might produce very different optimal solutions for different noise models used for the same problem. In general, researchers tend to model the noise according to a Gaussian model, but it could also be modeled according to non-Gaussian noise, such as Middleton noise [78] or Alpha-stable noise [79,80]. Moreover, one can see that the estimator in (2) is highly non-convex, presenting singularities at all of the true sensor positions. The single-cost-function optimization problem is thus an appropriate candidate for applying metaheuristic optimization methods, namely, swarm-based optimization.

**Figure 1:**
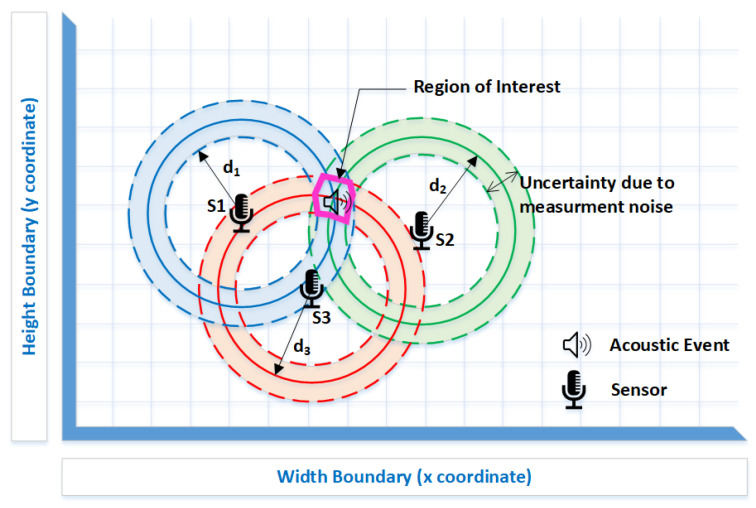
Geometry of the search space and measurement uncertainties.

When considering ideal conditions, the solution of the optimization problem would be a single point in the two-dimensional plane. This point would be the unique intersection of the circumferences’ radii, which would be centered on the sensors with distance $\hat{d_{i}}=\sqrt{g_{i}P/y_{i}}$ (represented by solid lines in Figure 1). Due to the measurement noise in the energy observations, an added or subtracted effect will distort the distance estimation, implying the appearance of two or more intersections, or perhaps even no intersections at all (represented by dashed lines in Figure 1). Hence, the solution of the optimization problem (2) lies in the region of interest (please see Figure 1), which is obtained by minimizing the sum of the squared difference between the observations and the measurement model (Equation (1)).

### 3.2. Swarm Intelligence

In the current section, an overview of swarm optimization is firstly presented by providing the general sequence of the steps that compose a typical swarm algorithm, with the specifics of each of the selected algorithms being described afterwards. It is worth mentioning that a general nomenclature is adopted rather than an algorithm-specific one (e.g., an agent is simply called an agent rather than coyote, wolf, or elephant, as used within the original methods). An overview of the nomenclature is provided in Appendix A.

**Figure 2:**
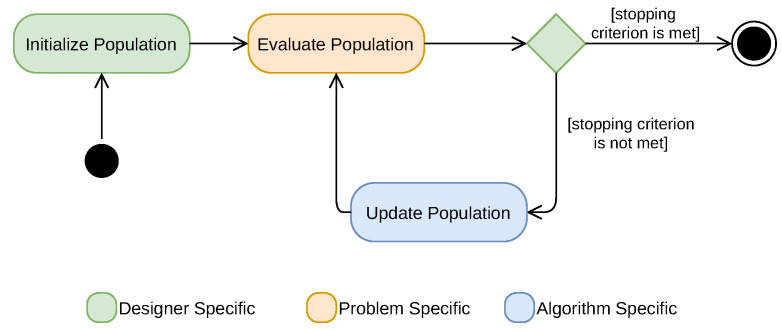
Activity sequence in swarm-based optimization.

Swarm intelligence algorithms have very similar activity sequences among them. The common steps are: (1) initializing the population, (2) evaluating the population (testing the cost function on the existent solutions of the population), (3) testing the stopping criterion, (4) updating the population (updating the position of the search agents in the search space), and cyclically repeating steps (2), (3), and (4) until a stopping criterion is met (please see Figure 2).

The population initialization can be considered a crucial step, since starting the search *far away* from the global optimum might prevent a method from finding the global solution [81]. In addition to generic methods, such as the pseudo-random number generator [82] or the chaotic number generator [83], initialization strategies specific to certain applications were also considered for particular problems, i.e., acoustic localization [55]. Random initialization is done by randomly spreading the agents throughout the search space. Typically, this process follows a uniform distribution bounded by the physical limits of the search space, such that each search agent has a random initial position $x^{0}∼U\left(\mathrm{lb},\;\mathrm{ub}\right)$, and lb and ub are vectors with the lower and upper bounds, respectively, for each dimension of the search space. This initialization method is used when no information about the problem is available at the initial phase or when that information should be ignored (e.g., in benchmarking when well-known functions are used (including their optimal solution(s)). However, when approaching a specific problem, it is common to have information that can be used to our advantage to initialize the agents. Smart or intelligent initialization means the determination of the areas of the search space where the best solution or solutions are expected to be and then initializing the search agents within those areas. For instance, for the EBAL problem, this can be done as explained in [55].

Once the agents in the population are initialized, their positions in the search space should be evaluated against the cost function so that in the end of the evaluation step, all agents have a cost value associated with them. After this, a stopping criterion is tested to see whether the obtained solutions are good enough, the algorithm has converged, a maximum number of function evaluations has been reached, or a combination of the three. If the stopping criterion is not met, the agents are moved within the search space in search of better solutions. The way the *movement*, *position update*, or *walk* is done is one of the features that should distinguish a swarm-based algorithm. The great diversity of nature is typically the inspiration for a wide variety of new update strategies. However, the mathematical models can be considered quite similar when cross-referencing some methodologies.

A transition of an agent’s position from iteration *t* to t+1 is usually defined by its current position $x^{t}$, a step direction s, and a step scale factor α, such that:

(3) $$x^{t+1}=x^{t}+\alpha ⊙s,$$

where ⊙ is the element-wise multiplication. The way α and s are calculated depends on the algorithm itself and involves some stochastic variables and the position of other agents. Each agent’s position is typically updated once or twice per iteration, following some variation of Equation (3).

In the following, we present the main particularities of each of the considered algorithms.

#### 3.2.1. Cuckoo Search

The CS algorithm, which was initially proposed by [16], arises from brood parasite species of cuckoos that lay their eggs in the nests of other specimens, expecting that other birds will take care of them.

At each iteration, there are two operations applied to all agents: random update (exploration) and discovery (exploitation).

From (3), the random updating operator can be defined as

$$x^{t+1}=x^{t}+\alpha \cdot s,\;\mathrm{with}\;\;\alpha =0.01\;\mathrm{and}\;\;s=\left(x^{t}-x_{*}^{t}\right)⊙R_{0}⊙R_{1},$$

where $R_{0}$ and $R_{1}$ are vectors of random values, such that $R_{0}∼N\left(0,1\right)$, $R_{1}∼L\left(1.5,1\right)$, and $x_{*}^{t}$ is the best search agent in the population at iteration *t*. The value of α is intended to avoid large flights that could easily make the agents jump outside the search space [84], whereas $\left(x^{t}-x_{*}^{t}\right)$ reduces the step length for agents closer to the best one, causing the best agent to stay at the same position.

After this updating operator, the discovery of fraction $p_{a}$ of the agents is done with

$$x^{t+1}=\mathrm{discovery}\left(x^{t}\right)=x^{t}+\alpha \cdot s⊙H\left(p_{a}-\epsilon \right)⊙\left(x_{r_{1}}^{t}-x_{r_{2}}^{t}\right),$$

where s is a vector of random values drawn from a standard normal distribution, such that s∼N(0, 1), *H* is the Heaviside function, $p_{a}=0.25$, ϵ∼U(0, 1), and $x_{r_{1}}^{t}$ and $x_{r_{2}}^{t}$ are two different agents selected randomly through random permutation [84].

Actually, both updates persist only if the new solution is better than the current one, i.e.,

$$x^{t+1}=\left\{\begin{array}{l} x^{t}+\alpha \cdot s,\;\mathrm{if}\;\;f\left(x^{t}+\alpha \cdot s\right)<f\left(x^{t}\right)\\ x^{t},\;\mathrm{otherwise} \end{array}\right.$$

for the random update operator, and

$$x^{t+1}=\left\{\begin{array}{c} \mathrm{discovery}\left(x^{t}\right),\;\mathrm{if}\;\;f\left(\mathrm{discovery}\left(x^{t}\right)\right)<f\left(x^{t}\right)\\ x^{t},\;\mathrm{otherwise} \end{array}\right.$$

for the discovery operator, where, in this case, *f* denotes the cost function, which should be minimized.

The CS then has an implicit strategy of greedy elitism for both updating operators, where the quality of an agent never degrades.

#### 3.2.2. Grey Wolf Optimizer

The GWO algorithm was proposed in [61] and was inspired by the hunting behavior of gray wolfs and their social hierarchy. In this optimization procedure, the agents are wolves chasing the prey (optimal solution); however, because the optimal solution is unknown, it is considered that the wolves at the top of the hierarchy have better knowledge about the location of the prey and are closer to it [61]. Thus, the procedure is based on a simple updating operator that considers the position of the three best agents (the agents whose positions correspond to the best solutions), which are known, respectively, as alpha, beta, and delta (in descending order of the solution quality).

The updating operator in GWO is different from the general concept of (3), and the next location of each agent is given by

$$x^{t+1}=\frac{s_{\alpha }+s_{\beta }+s_{\delta }}{3},$$

where the $s_{e}$ vectors are calculated as

(4) $$s_{e}=x_{e}^{t}-R_{0}⊙\left(\right|R_{1}⊙x_{e}^{t}-x^{t}\left|\right),\;\forall e\in \left\{\alpha ,\beta ,\delta \right\},$$

where $x^{t}$ is the agent’s current position; $x_{\alpha }^{t}$, $x_{\beta }^{t}$, and $x_{\delta }^{t}$ are the positions of the alpha, beta, and delta wolves, respectively, $R_{0}∼U\left(-a,a\right)$, and *a* is linearly decreased from 2 to 0 over the course of the iterations such that $R_{0}∼U\left(-2+t/t_{max},2-t/t_{max}\right)$, where *t* is the current iteration, $t_{max}$ is the maximum number of iterations, and $R_{1}∼U\left(0,2\right)$.

The goal of the decrease in *a* over the course of the iterations is to control the trade-off between exploration and exploitation. As *a* decreases, $R_{0}$ tends to assume values closer to 0, which diminishes $|R_{1}⊙x_{e}^{t}-x^{t}|$ in (4), which, in turn, forces the new solution to converge to $x_{\alpha }^{t}$, $x_{\beta }^{t}$, and $x_{\delta }^{t}$. As such, exploration is favored in the initial iterations, while exploitation is favored in the latter.

#### 3.2.3. Enhanced Elephant Herding Optimization

The EHO method, which was originally proposed in 2015 [51], was inspired by elephants’ herding behavior, where a group of elephants, mainly calves and females, follow a matriarch, thus forming a clan of elephants. The algorithm also considers the fact that male elephants may leave the clan to live alone when they reach adulthood. Accordingly, the paper in [51] presents a multi-population algorithm where the population is divided into several groups of agents by applying two operators: a clan updating operator, in which the agents tend to move towards the best agent of their group, and a separating operator, where some agents are repositioned randomly in the search space, mimicking desertion from the group.

Aside from the inspiration, three major drawbacks in the EHO algorithm were identified in [69]—unjustified convergence towards the search space’s origin in the matriarch update operator, unbalanced exploration/exploitation trade-off in the group updating operator, and skewed agent distribution in the separating operator. Hence, the evolution of the EHO into the EEHO was proposed in [69] with better performance in benchmarking than that of the EHO. The EEHO algorithm is not just an improvement over the EHO, but a rectification of relevant problems that cannot be ignored.

In the EEHO method, the clan updating operator updates each agent $x_{c}^{t}$ of each group *c* with a combination of three steps:

(5) $$x_{c}^{t+1}=x_{c}^{t}+\alpha s_{1}+\beta s_{2}+\gamma s_{3},\;\mathrm{with}\;\;s_{1}={x_{c}^{t}}_{*}-x_{c}^{t}\;\mathrm{and}\;\;s_{2}=c_{c}^{t}-x_{c}^{t},$$

where ${x_{c}^{t}}_{*}$ is the best agent in the group *c*; $c_{c}^{t}$ is the center of group *c*, which is obtained by averaging the position of the agent in group *c*; $s_{3}∼U\left(-\left(\mathrm{ub}-\mathrm{lb}\right),\left(\mathrm{ub}-\mathrm{lb}\right)\right)$; α, β, and γ, are, respectively, the best agent, the group center, and the randomized influence factors, respectively. The position of the best agent in group *c*, ${x_{c}^{t}}_{*}$, is not updated as in (5), but instead as:

$$x_{c*}^{t+1}=x_{c*}^{t}+\beta s_{2},\;\mathrm{with}\;\;s_{2}=c_{c}^{t}-x_{c*}^{t},$$

where, once again, β is the influence factor of the group center.

After the clan updating operator is executed, the worst agents in each group are randomly repositioned in the search space with

$$x_{c}^{t+1}=R,\;R∼U\left(\mathrm{lb},\mathrm{ub}\right),$$

with the possibility of finding new local optimums (exploration).

#### 3.2.4. Moth–Flame Optimization

The MFO algorithm was proposed in [63] in 2015. Its inspiration comes from a navigational strategy that some flying insects use to move in a straight line over a given time. In particular, at night, moths fly by maintaining a fixed angle with respect to a source of light [63]. This strategy only works if the source of light is a further distance away than the traveled distance, since it will otherwise lead to circular flying around the source of light. Before human-made artificial lights, the moon was the reference that moths used to guide their flying, and the result was a straight path. However, nowadays, moths easily get trapped in artificial sources of light, such as lamps, and fly around them indefinitely. This circular behavior around a source of light is actually what is mimicked.

In the MFO, the light sources are considered plausible optimum solutions, and the moths search around them. To improve exploration and to avoid falling into one local optimum, several light sources are considered, and each moth updates its position with regard to one of these lights at each iteration. Thus, the position update of each search agent (moth) between iterations *t* and (t+1) is defined as

(6) $$x^{t+1}=x_{ls}^{t}+\left|x_{ls}^{t}-x^{t}\right|⊙\exp\left(bR\right)⊙\cos\left(2\pi R\right),$$

where *b* is the spiral-shape constant; $x_{ls}^{t}$ is the position of a chosen light source; R∼U(−r,1) is a vector of random numbers; *r* decreases linearly from −1 to −2 over the course of iterations.

The light sources represent the best solutions found up to a moment and are updated in every iteration if new and *better* solutions are found. In order to increase exploitation over the course of the iterations, the number of light sources is also reduced, forcing the agents to update their positions with respect to the same sources of light, i.e., forcing the algorithm to converge. Let $N_{ls}$ be the number of light sources; then, at iteration *t*,

$$N_{ls}=\left\lfloor N_{ls}^{0}-\frac{t}{t_{max}}\left(N_{ls}^{0}-1\right)\right\rceil ,$$

where ⌊•⌉ means rounding to the nearest integer, $N_{ls}^{0}$ is the initial number of light sources (typically equal to the number of agents), and $t_{max}$ is the maximum number of iterations.

The value *r*, which decreases linearly from −1 to −2 over the course of the iterations, can also be defined in a similar way with

$$r=-1-\frac{t}{t_{max}}.$$

It can be seen from (6) that when R assumes negative values, the agent gets closer to the light source, whereas when it assumes positive values, the agent moves away from the light source. Therefore, while $N_{ls}$ controls the number of local search areas, *r* tries to control the scattering of the search agents around those areas. With different strategies, they both try to control the trade-off between exploration and exploitation over the iterations (favoring exploration initially and exploitation afterwards).

#### 3.2.5. Whale Optimization Algorithm

The WOA (proposed in 2016 [64]) was inspired by the bubble-net hunting behavior of humpback whales. The WOA applies to each agent’s (whale’s) position one of three possible updating operators at each iteration. Two of these operators are very similar to the one used by the GWO algorithm (Section 3.2.2). These are

(7) $$x^{t+1}=s_{e},\;\mathrm{for}\;e=*$$

and

(8) $$x^{t+1}=s_{e},\;\mathrm{for}\;e=rand$$

where $s_{e}$ is calculated exactly as in (4), $x_{*}^{t}$ is the position of the best agent in the current iteration, and $x_{rand}^{t}$ is the position of a random agent from the current population.

The third operator, called the spiral updating operator, is borrowed from the MFO algorithm (Section 3.2.4, (6)), but, instead of using a light source as a reference, the best whale is used, such that

(9) $$x^{t+1}=x_{*}^{t}+\left|x_{*}^{t}-x^{t}\right|⊙\exp\left(bR_{2}\right)⊙\cos\left(2\pi R_{2}\right),$$

where *b* is once again the spiral-shape constant; $R_{2}$ is a vector of random values such that $R_{2}∼U\left(-1,1\right)$; $x_{*}^{t}$ is the position of the best agent.

As previously mentioned, at each iteration, each agent updates its position with only one of these three operators. Hence, for each agent, the choice is made as shown in Algorithm 1, where *a* decreases linearly over the course of the iterations, with the same goal as in GWO (to control exploration and exploitation).
**Algorithm 1** Selection of the updating operator.Generate p∼U(0,1) **if**
p<0.5
**then**
    Generate $R_{0}∼U\left(-a,a\right)$ (Section 3.2.2)
    **if** $|R_{0}|<1$ **then**

        Update agent with (7). (exploitation)

    **else**

        Update agent with (8). (exploration)

    **end if**

**else**

    Update agent with (9)

**end if**


As a concluding remark, it is clear that the WOA is a conjunction of both the GWO and MFO, i.e., it can be considered as an integration of the two algorithms.

#### 3.2.6. Salp Swarm Algorithm

The SSA was proposed in [65], and its inspiration came from the collective behavior of salps that group together alongside another, arranged in a chain.

To simulate the salp chain, the SSA proposes a food source as the search goal, a chain leader that guides the movement towards the food, and the salp followers, who follow one after the other after the leader. The food source is the best solution found so far, $x_{*}$, and the leader is the best search agent in the current iteration *t*, $x_{*}^{t}$. Then, Ref. [65] proposed updating the leader’s position as

(10) $$x_{*}^{t+1}=x_{*}+\alpha \cdot R_{0}⊙\left(R_{1}⊙\left(\mathrm{ub}-\mathrm{lb}\right)+\mathrm{lb}\right),$$

where $R_{0}$ is a vector of values equal to −1 or 1 with equal probabilities, i.e., $P\left({R_{0}}_{j}=-1\right)=P\left({R_{0}}_{j}=1\right)=0.5$ for each dimension *j* of $R_{0}$, $R_{1}∼U\left(0,1\right)$ is a vector of random values, ub and lb are vectors with the upper and lower bounds of the search space, respectively, and α is a coefficient that balances exploration and exploitation by decreasing its value over the course of the iterations according to

$$\alpha =2\exp(-{\left(4t/t_{max}\right)}^{2}),$$

where *t* is the current iteration and $t_{max}$ the maximum number of iterations.

Because each agent in the followers’ group should follow one after the other, the *i*th agent in the population is updated with:

(11) $$x_{i}^{t+1}=\frac{1}{2}\left(x_{i}^{t}+x_{i-1}^{t}\right),\;\mathrm{for}\;\;i=2,3,\ldots ,n,$$

where *n* is the population size and $x_{1}^{t}$ is the position of the best agent at iteration *t*.

The facts that (1) only one of the *n* individuals in the population updates its position using stochastic values and (2) all the others just follow the leader one after the other might suggest that the algorithm does not perform very well. Indeed, to improve the performance, the original MATLAB^®^ implementation updates one half of the population according to (10) and the other half according to (11). The same was done in the developed implementation.

#### 3.2.7. Tree Growth Algorithm

The TGA was proposed in 2018 and was inspired by the way that trees grow depending on their prioritized needs (light or soil resources) [66]. The TGA presents four operators to be applied on four groups in the population (Table 3). After sorting the search agents according to their quality (with the best agent first) to the best $N_{1}$ trees (search agents), a local search operator is applied, where the new position of the search agent only depends on its current position and on stochastic variables. The best $N_{2}$ trees after the best $N_{1}$ trees are called competition trees, and for these, the updating operator considers the position of some trees in the best tree group, the current position of the tree to be updated, and some stochastic variables. After the competition group, the $N_{3}$ search agents are randomly repositioned in the search space. The same happens to the $N_{4}$ search agents after these, but after the repositioning, their position-vector dimensions are mixed with the ones in the position vector of the best tree in the population.

The first operator, applied to the trees in the best tree group, is defined as:

(12) $$x^{t+1}=\frac{x^{t}}{\theta }+R⊙x^{t},$$

where R∼U(0, 1) is a vector of random values, and θ is a constant value.

The second updating operator, which is applied to the competition trees, is defined as:

(13) $$x^{t+1}=x^{t}+R_{0}⊙\left(\lambda x_{0}+\left(1-\lambda \right)x_{1}\right),$$

where $R_{0}∼U\left(0,\;1\right)$ is a vector of random values, λ is also a constant value, and $x_{0}$ and $x_{1}$ are the positions of the two search agents from the best tree group closest to $x^{t}$.

The third updating operator, which is applied to the removed trees, is defined as

(14) $$x^{t+1}=R,$$

where R∼U(lb, ub) is a vector of random values.

The fourth updating operator, as stated above, results in new trees, where each dimension value is either equal to a dimension value of the best tree in the population or equal to a random value within the search space’s bounds. This operator is applied to the reproduction group, and it can be defined as:

(15) $$x^{t+1}=R_{1}⊙R_{0}+\left(1-R_{1}\right)⊙x_{*}^{t},$$

where $R_{0}∼U\left(\mathrm{lb},\;\mathrm{ub}\right)$ is a vector of random values within the search space, $R_{1}$ is a bit vector of values equal to 0 or 1 with equal probability, and $x_{*}^{t}$ is the position of the best search agent in all of the population in the current iteration *t*.

#### 3.2.8. Coyote Optimization Algorithm

The COA is a swarm-based algorithm that was proposed in 2018 and was inspired by coyotes’ social behavior [67]. Similarly to the EHO, it is a multi-population method, meaning that the entire population is divided into independent sub-populations or groups, which are called packs, referring to coyote groups.

Once the coyotes (search agents) are divided into packs, each agent of pack *p* is updated by following a variation of (3):

$$x_{p}^{t+1}=x_{p}^{t}+R_{1}⊙s_{1}+R_{2}⊙s_{2},\;\mathrm{with}\;\;s_{1}=x_{p*}^{t}-x_{p}^{t}\;\mathrm{and}\;\;s_{2}=M_{p}-x_{p}^{t},$$

where $R_{1}∼U\left(0,\;1\right)$ and $R_{2}∼U\left(0,\;1\right)$, $x_{p*}^{t}$ is the best agent in pack *p*, and $M_{p}$ is the median of the search agents’ positions in pack *p*.

After all agents in a pack are updated, a new search agent (called a pup) is generated by a random combination of dimension values of other agents or random values in the search space. If some agents in the group have higher costs than the pup, the oldest of these is replaced by the new pup; otherwise, the pup dies.

After this, a last operator is applied before the iteration ends; it exchanges agents between groups with some probability of increasing the diversity inside the groups. Finally, the *ages* of all agents are incremented and the iteration is concluded.

#### 3.2.9. Supply–Demand Optimization

The SDO algorithm, which was proposed in [68], gained its inspiration from a set of fundamentals in economic theory concerning commodity prices and quantities in markets, and it states that these two values might have periods of instability (where they tend to fluctuate) and stability (where they tend to an equilibrium point) [68].

Based on this, SDO divides the initial population into two sub-populations (or groups) of equal sizes, which are called the quantities (*Q*) and the prices (*P*). Each quantity has an associated price (and vice-versa), such that each search agent in *Q* has a corresponding search agent *P*. At each iteration *t*, the “equilibrium” quantity ($x_{Q_{eq}}^{t}$) and price ($x_{P_{eq}}^{t}$) are defined based on the current solutions. The value of $x_{Q_{eq}}^{t}$ is defined by roulette-wheel selection [68] from the search agents in population *Q*, and the best search agents have a higher probability of being selected. The price $x_{P_{eq}}^{t}$ is defined by roulette-wheel selection half of the time (from population *P*), and in the other times, it is defined by the average of the positions of the search agents in *P*.

Once $x_{Q_{eq}}^{t}$ and $x_{P_{eq}}^{t}$ are defined, the search agents from *Q* (quantities) are updated:

$$x_{Q}^{t+1}=x_{Q_{eq}}^{t}+\alpha \cdot \left(x_{P}^{t}-x_{P_{eq}}^{t}\right),$$

where $x_{P}^{t}$ is the corresponding search agent in *P* (price), and α is defined as:

$$\alpha =2\cdot \frac{t_{max}-t-1}{t_{max}}\cdot \sin\left(2\pi r\right),$$

where *t* is the current iteration, $t_{max}$ is the maximum number of iterations, and r∼U(0,1) is a random number.

The agents in group *P* are updated with:

$$x_{P}^{t+1}=x_{P_{eq}}^{t}+\beta \cdot \left(x_{Q}^{t+1}-x_{Q_{eq}}^{t}\right),$$

where $x_{Q}^{t+1}$ is the corresponding search agent in group *Q*, and β is defined as:

β=2·cos(2πr),

where r∼U(0, 1) is a random number redefined at each iteration.

The original paper states that whenever the new price $x_{P}^{t+1}$ is better than the quantity $x_{Q}^{t+1}$, the quantity should be replaced by the price. However, this causes a loss of diversity without a gain in intensification, and it is just doubling a solution. Instead of that, the original MATLAB^®^ implementation (as well as the implementation used in this work) does not replace the quantity with the price if the latter is better, but only updates the new solutions (either $x_{P}^{t+1}$ and $x_{Q}^{t+1}$) if it means an improvement regarding the objective function, meaning that the costs of the solutions never get worse, as it is for the CS algorithm (Section 3.2.1).

#### 3.2.10. Momentum Search Algorithm

The MSA was published in the year 2020. Inspired by the momentum conservation law [72], it can be considered as both a physical and a swarm-based algorithm. In the MSA, each solution, or search agent, is a body with a mass *m* proportional to its quality, such that, at each iteration *t*,

$$m^{t}=\frac{f\left(x^{t}\right)-f\left(x_{worst}^{t}\right)}{f\left(x_{best}^{t}\right)-f\left(x_{worst}^{t}\right)}.$$

Then, at each iteration, an external body collides once against each of the search agents, moving each towards the heaviest body (the best solution).

The momentum of this external body is the key point in the MSA for controlling the trade-off between exploration and exploitation. When the external body collides at a higher momentum, the other bodies will change their positions more radically. When the momentum is lower, the other bodies will experience small position updates.

The momentum p of a body depends on its mass and velocity, and it is defined as:

p=mv.

As such, to calculate the momentum of the external body, its mass and velocity need to be known. The mass of the external body at iteration *t* is defined as:

$$m_{ext}^{t}=1-\frac{t-1}{t_{max}-1}$$

and its velocity before each collision against a search agent $x^{t}$ is defined as:

$$v_{ext}^{t}=\left(1-\frac{t-1}{t_{max}-1}\right)\cdot R⊙v_{max}\cdot \mathrm{sgn}\left(x_{best}^{t}-x^{t}\right),$$

where $t_{max}$ is the maximum number of iterations, R∼U(0, 1) is a vector of random values, sgn is the sign function, and $v_{max}$ is a constant value representing the maximum possible speed.

Finally, by the momentum conservation law (more details in [72]), the velocity of each search agent after the collision at iteration *t* can be calculated as:

$$v^{t}=\frac{2m_{ext}^{t}}{m^{t}+m_{ext}^{t}}v_{ext}^{t}.$$

Then, the position $x^{t}$ is updated with:

$$x^{t+1}=x^{t}+R⊙v^{t}.$$

#### 3.2.11. Summary

Having seen the algorithms individually and in detail, it is possible to recognize some common features and others that might differentiate them. As a major difference in this group of methods, one can see that some methods divide the whole population into independent sub-groups, while others do not; this property improves the exploration phase over exploitation. As such, this property might be of great importance when searching for more complicated, highly non-convex spaces. Another feature concerns the distribution of random variables employed in the algorithms. From the presented methods, only Cuckoo Search relies on non-uniform stochastic variables, namely, on normal and Lévy ones. In unbounded search spaces, the Lévy flight behavior might offer outstanding exploration capacities to algorithms; however, when the space’s bounds are known, it might be sufficient (and more efficient) to rely only on uniform random variables. Starting from the Grey Wolf in 2014, many algorithms have started to use an exploration/exploitation strategy that depends on a predefined maximum number of iterations. This feature allows the algorithms to begin with a strong stage of exploration of the search space that transitions to a strong exploitation stage in the last iterations. This feature should always be considered if one wants an algorithm to run a fixed and known number of iterations. Lastly, there is a property related to how the population’s quality can evolve over iterations or generations. Most algorithms use an elitism strategy, where only the k best individuals are preserved and passed directly to the next generation. At the same time, the remaining ones are subject to operators that might improve their fitness, but might also deteriorate it. Other algorithms, however, have a much greedier behavior where the operators are applied to every search agent, but the resulting mutations are only preserved if the agent improves its quality and are reverted otherwise. This greedy behavior favors exploitation by clearly sacrificing the exploration capacity. Table 4 summarizes these four properties in the selected algorithms. Please note that the presented properties are not general indicators of the performance of the algorithms, since performance is always dependent on the problem to which the algorithms are applied.

### 3.3. Population Initialization

A common feature of the analyzed algorithms is the fact that they all depend on the computation of an initial population. Over recent years, initialization techniques have attracted much attention in the research community, which is in search of constant improvements [85]. One of the simplest and most widely used methods is randomization, the aim of which is to produce evenly distributed populations [81]. The initialization step is critical in population generation because it not only because it can improve the convergence rate of an algorithm, but unsatisfactory preliminary guesses can also possibly lead the search away from optimal solutions. Apart from generic techniques, such as a pseudo-random number generator [86] or chaotic number generator [87], there are initialization schemes that are particularly designed for a specific type of problem, such as the ones for antenna design [81] or image segmentation [88].

Since, most commonly, the agents are deployed adrift over the search region with no prior consideration of any particularities of the problem of interest, it is very hard to achieve any kind of progress. Therefore, it is better that one bears in mind all additional information about the problem, such as knowledge about the employed observation model, and that one uses it as leverage to produce better starting points. This could be done, for instance, by taking advantage of the acoustic decay model in (1), from which a distance estimate between a sensor $s_{i}$ and the source can be obtained from $y_{i}$ according to

(16) $$\hat{d_{i}}=\sqrt{\frac{g_{i}P}{y_{i}}},\;i=1,\ldots ,N.$$

The distance obtained from (16) represents an ML estimate of the distance between the source and the *i*-th sensor. This simply tells us that the source lies within the circle (in 2-D) centered at the *i*th sensor with a radius equal to $\hat{d_{i}}$. Since, as mentioned in Section 3.1, there will not be a unique intersection point, when considering a pair of measures, several situations can arise, namely, secant circumferences and external or internal circumferences. The methodology for creating the initial population of agents considers the center of the convex hull formed by the intersection of pairwise measures [55] for secant circumferences or the middle point of the straight-line segment between pair-wise sensors. For further details, please refer to [55].

## 4. Testing Procedure and Experimental Setup

Regarding the implementation, the selected algorithms were implemented in the C language, with the original publications and associated MATLAB^®^ source code (when available) serving as the basis. In the end, since the algorithms have several similarities between them and, in the optimization procedure, only parts are algorithm-specific (Figure 2), most of the code written—cost function, mathematical operations, main data structures, and initialization and stopping criteria—was shared between the different algorithms. The test script for obtaining the simulation results shown in this work was conceptually equal to the one published in [89], which was based on a MATLAB^®^ script that repeatedly sends energies to an embedded device and receives the estimated location and associated statistics. However, as the Raspberry boards had more persistent memory than the ones used in [89], here, it was possible to preload a batch of energies on the board and then have a C-language script doing the testing of the control flow. Since this was done on-board with compiled code, the simulations could be done in a reduced time. The testing procedure on the board is detailed in Algorithm 2, where the input datasets and file results (both in JSON format) were generated and analyzed, respectively, in the MATLAB^®^ environment.

As already stated, in this study, three main goals were considered: (1) comparing the performance of the selected algorithms in solving the EBAL problem, (2) validating whether the smart/intelligent initialization improved their accuracy, and (3) analyzing the feasibility of the selected methods for running on computationally low-power devices. For the first two goals, the cost and the error ($||x-\hat{x}\left|\right|$) of the best agent found so far over the algorithms’ iterations were analyzed. For the third goal, the simulation execution time was considered. Thus, for each simulation, *t* agents/solutions and the simulation execution time were recorded (where *t* is the number of iterations necessary for the algorithm to reach the maximum number of function evaluations).
**Algorithm 2** Testing procedure on the Raspberry boards.
N={6,9,12,15}▹ Number of sensors
V={−80,−75,−70,−65,−60,−55}▹ Noise variances
I={Random,Intelligent}▹ Types of swarm initialization
A={CS,GWO,EEHO,MFO,WOA,SSA,TGA,COA,SDO,MFO}
**for all**(n,v,i,a)∈N×V×I×A**do**
    data=LoadDataset(n,v) ▹ Energies, sensors’ positions, etc.    results=[]
    **for** m=1,2,3,…,10.000 **do**
        startTime= Clock()
        $\hat{X}$ = Execute(a,n,i, data[*m*]) ▹ Location estimation by algorithm *a*. $\hat{X}$ contains the best solutions found so far at each iteration of the algorithm
        executionTime=Clock() − startTime
        $results=[results;\;\;\hat{X}\;\;executionTime]$
    **end for**
    SaveToFile(results)
**end for**


The energies generated ($y_{i}$) were corrupted by white Gaussian noise, ν, of variance $\sigma_{\nu }^{2}$ to approximate real situations. With the purpose of the extrapolation of the obtained results, different sets of sensors (N=6, N=9, N=12, and N=15) and variances (from $\sigma_{\nu }^{2}=-80$ dB to $\sigma_{\nu }^{2}=-55$ dB in intervals of 5 dB) were considered in a virtual search space with dimensions of 50 m × 50 m. For more reliable results, for each combination of the number of sensors, variance, algorithm, and initialization procedure, 10,000 Monte Carlo runs were executed, meaning that a total of 4,800,000 simulations were carried out. It should be noticed that, for each combination of sensors sets and variances, only one input dataset with 10,000 testing scenarios was generated and used by all different algorithms. This means that all algorithms were subject to the exact same scenarios. The transmitted power, gains, and decay propagation factor considered were set to P=5, $g_{i}=1$, and $\beta_{L}=2$, respectively. With the purpose of providing a benchmark for the comparison of the implemented algorithms, an exhaustive Grid Search method with 0.1 m of grid spacing was implemented (also in the C language) and tested in the same simulation conditions. Table 5 summarizes the model and the testing scenario parameters considered in the tests.

A fixed maximum number of function evaluations was used as the stopping condition for all tests. For all algorithms to evaluate the cost function exactly the same number times without interrupting any iterations, it was necessary to find the least common multiple of $\frac{Evaluations}{Iteration}$ between all of them. This value, or a multiple of it, could be used as the maximum number of evaluations. In the tests employed, 6000 function evaluations were performed in every test. The chosen value was sufficient for the convergence analysis (as will be shown in the next section, the optimization should not exceed one or two thousand function evaluations if a good algorithm and stopping criterion are used). Table 6 summarizes the overall parameters used for each method.

Some algorithms rely on random numbers that follow normal or Lévy symmetrical stable distributions. To generate those values, the Box–Muller method [90] and Mantegna’s algorithm [91] were used, respectively.

## 5. Results and Discussion

Two important performance metrics are the function cost, which is calculated with (2), and the error, i.e., the distance between the estimated location and the real (unknown) location. The correlation between these two variables is central in the approach to the EBAL problem used here, where the true goal is to reduce the error, but, because it is unknown, an estimated cost is considered and minimized. In ideal conditions, this correlation would be perfect, such that

$$\mathrm{if}\;\mathrm{cost}\left(x^{\prime }\right)<\mathrm{cost}\left(x^{\prime \prime }\right)\;\mathrm{then}\;\mathrm{error}\left(x^{\prime }\right)<\mathrm{error}\left(x^{\prime \prime }\right),\;\mathrm{for}\;\mathrm{all}\;x^{\prime },x^{\prime \prime }\in R^{2}$$

With this, a minimum value of the estimated cost would always mean a minimal error; however, there are two other independent variables that influence this correlation. The main one is the noise: As noise increases, the correlation between the estimator cost and the corresponding true error becomes unreliable, since noise perverts the measured energies considered in the estimator. Another variable, which is not as relevant as noise, is the number of measured energies (or sensors) considered in the estimator. Because the expected noise mean is null, considering more energies in the cost function might improve the correlation in a way in which individual errors might cancel each other out. Obviously, in a situation where the noise variance would be null, the number of energies would not matter. However, as the variance increases, the number of energies considered becomes more important. As such, caution should be taken when analyzing the correlation between the cost and the error in tests with higher noise values, mainly when a low number of sensors is considered.

Before analyzing the convergence plots in the next subsections, something should be clarified about the difference in the starting points of these convergence curves. The tested swarm algorithms have different population sizes, which means that the initialization also generates different numbers of initial solutions. When more solutions are generated, more diversity exists; thus, the best solution from those is likely to be better than the best one from a smaller set of generated solutions (the same applies to the worst solution: It is expected to be worse than the worst from a smaller set). That is why the convergence plots start at different cost values—methods with higher population sizes tend to have a better best initial solution, as well a worse worst solution, but, because the convergence plots only consider the best solution found so far, the convergences of these methods are expected to start at lower cost values. The CS algorithm, for instance, which has the lowest population size of all methods, is expected to have a convergence plot that starts above all of the plots of the other methods.

Bearing this in mind, the next three subsections compare the algorithms’ convergences (Section 5.1) and analyze the performance gains with smart/intelligent initialization (Section 5.2), as well as the computational times obtained on several embedded processors (Section 5.3).

### 5.1. Algorithm Comparison

**Figure 3:**
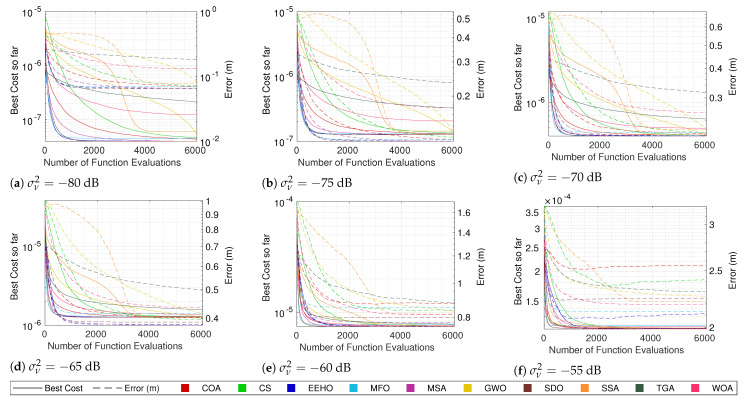
Cost convergence and respective error for each algorithm when N=6.

**Figure 4:**
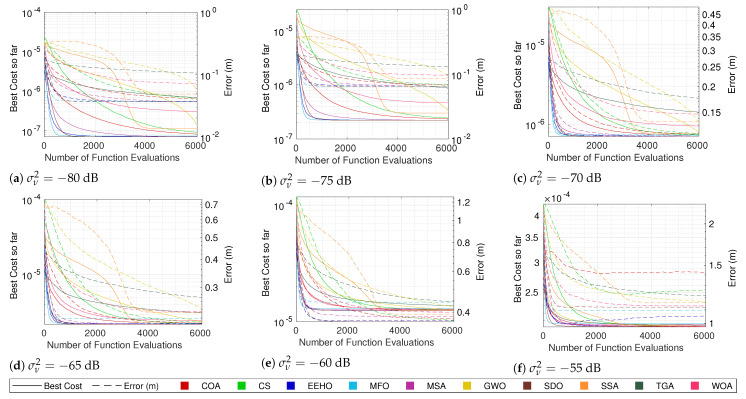
Cost convergence and respective error for each algorithm when N=9.

**Figure 5:**
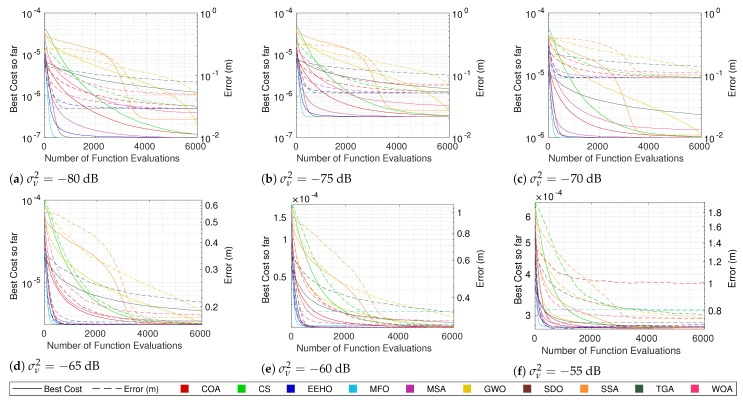
Cost convergence and respective error for each algorithm when N=12.

**Figure 6:**
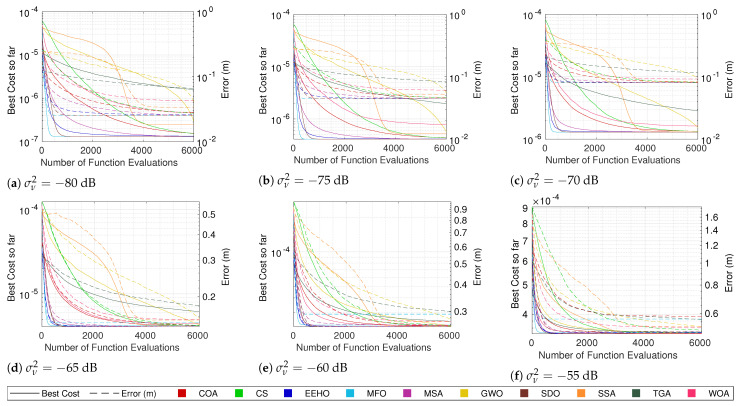
Cost convergence and respective error for each algorithm when N=15.

The cost convergence and respective error while using different algorithms with smart/intelligent initialization for different combinations of the number of sensors and noise are shown in Figure 3, Figure 4, Figure 5 and Figure 6. The plotted lines are the result of averaging 10,000 Monte Carlo runs. The continuous lines represent the averaging cost of the best solution in the current iteration, and the dashed lines represent the averaging error of that best solution. Since all of the tested optimization methods have some elitism strategy, the best solution in the current iteration is also the best solution found so far over all iterations in the optimization procedure (the reason for why the cost convergence curves are all always decreasing).

Because noise distorts the correlation between the cost and true error, the fact that the cost plots are always decreasing does not imply that the error plots are as well. In fact, it is possible to see in some plots (mainly the ones with higher noise values) that even the average error can increase at some moments. Nonetheless, in most cases, a strong correlation between the cost and the error can be seen, where the decrease in the cost is reflected by an error decrease, resulting in the methods that better minimize the cost function being the ones that get lower errors.

Observing the continuous lines, the three methods that converge the fastest towards the optimum are MFO, SDO, and EEHO, while methods such as the TGA, GWO, and SSA present the worst performances. Looking at the dashed lines (and as expected), it is possible to see that MFO, SDO, and EEHO are also the ones that achieve lower errors, while the TGA, GWO, and SSA present higher errors. For comparison, the mean error of the Grid Search in Table 7 shows that, while being an exhaustive search method that evaluates the cost function 501×501= 251,001 times, it achieves very similar accuracy to that of the swarm algorithms, which only evaluate the cost function 6000 times (with some convergence much before the 6000 function evaluations).

It should be noted that the late convergence of methods such as GWO and SSA is due to their native exploration and exploitation control strategies, which depend on the maximum number of iterations. In GWO, this is even more problematic with lower noise because, as the end of the curve in Figure 6a shows, it reaches the stopping criteria before fully converging (increasing the maximum number of iterations does not change this issue).

### 5.2. Smart/Intelligent vs. Random Initialization

The present section intends to provide an understanding of the impact of the smart/ intelligent initialization proposed in [55] on the performance of the different algorithms that were implemented. It was already shown that it improves the performance of the EHO in terms of both cost and localization error [55]. Now, it will be shown whether or not this initialization can be generalized to any swarm-based optimization algorithm.

**Figure 7:**
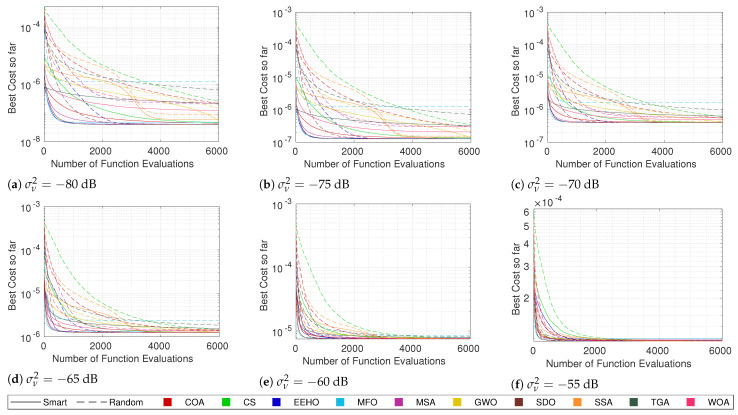
Cost convergence when using smart and random initialization for each algorithm when N=6.

**Figure 8:**
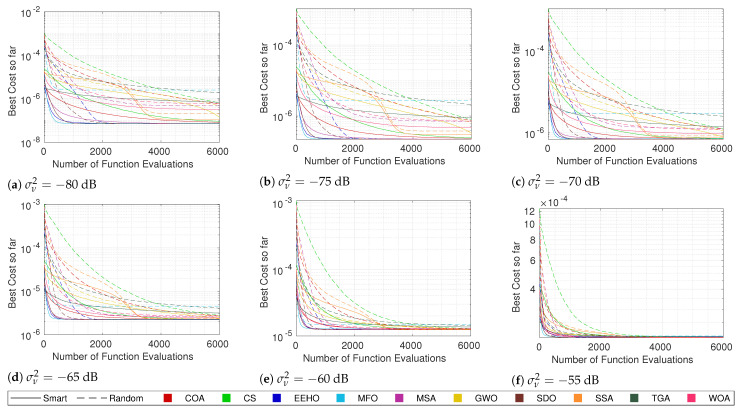
Cost convergence when using smart and random initialization for each algorithm when N=9.

**Figure 9:**
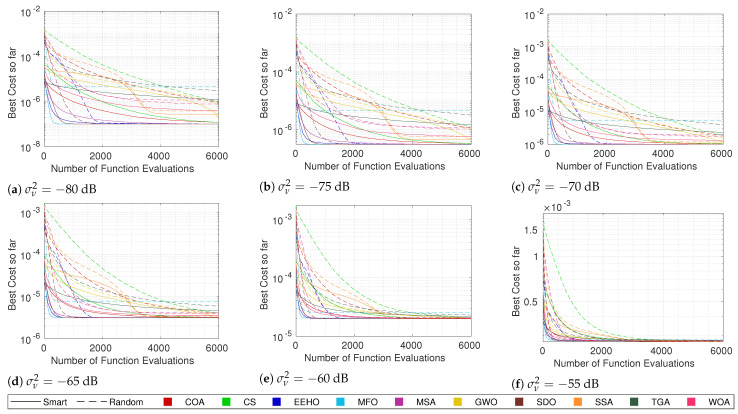
Cost convergence when using smart and random initialization for each algorithm when N=12.

**Figure 10:**
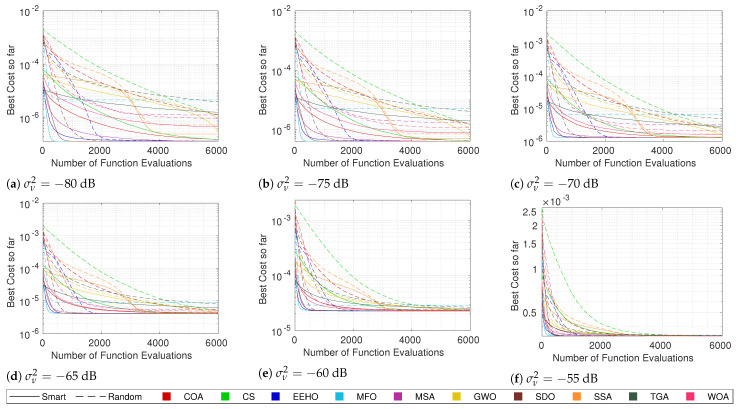
Cost convergence when using smart and random initialization for each algorithm when N=15.

For this purpose, the same tests that were performed in the previous section were carried out, but using random initialization. Since it is already known that, generally, there is a strong correlation between the cost and the error, the focus will just be on the cost convergence of the different methods when using both types of swarm initialization. Figure 7, Figure 8, Figure 9 and Figure 10 compare the cost convergences of the different methods when using smart/intelligent initialization (continuous lines) and when using random initialization (dashed lines) for different combinations of numbers of sensors and noise.

Since the smart/intelligent initialization generates the initial solutions in a reduced search area in which it is believed that the global optimum lies, it is obvious that, when using smart initialization, the best initial costs are, on average, much lower than when using random initialization. Because of this, it is possible to see that the dashed lines (random initialization) all start above the continuous lines (smart/intelligent initialization). Moreover, no dashed line of any algorithm crosses the respective continuous line at any moment throughout the iterations, which means that, on average, using smart initialization is always better than or equal to using a random initialization. The term “equal” is justified because, as can be seen, for example, in Figure 10a, SDO and EEHO using random initialization can reach, on average, the same optimums that they reach when using smart initialization (their dashed lines join the respective continuous lines at around 2000 function evaluations). Nevertheless, these are the only methods where the benefits of smart initialization stop before 6000 function evaluations. At 6000 function evaluations, none of the other algorithms have yet (on average) reached the same optimums as when using the smart/intelligent initialization. With this, it can be seen that smart/intelligent initialization not only works for any swarm algorithm, but it is even more relevant for most of them than it is for EEHO.

It is interesting to see that with random initialization, in contrast with what happens when using smart/intelligent initialization, methods with stronger initial exploration phases, such as GWO, the WOA, and the SSA, outperform methods such as the COA and CS, which do not employ special care for the initial exploration techniques. Nonetheless, with the exception of SDO and EEHO, it possible to see a tendency for the methods to stagnate in sub-optimal solutions when using random initialization. These two facts imply that smart/intelligent initialization, by initializing the population in a restricted area in which the global optimum is believed to be, not only facilitates the optimization task for methods that have weak exploration operators, but also avoids that methods with strong initial exploration phases get trapped in sub-optimums that are far away from the global optimum, as seems to happen with MFO, the MSA, and the SSA when using random initialization.

### 5.3. Time Efficiency

It is known that a key feature of swarm-based algorithms is their low computational complexity. However, this low complexity is not sufficient for knowing a priori exactly how time-consuming these algorithms are when solving the EBAL problem in real applications. In the same way, the widely available test benches implemented in MATLAB^®^ and executed on powerful computational platforms are not sufficient, since it is impossible (or not very feasible) to have those processing cores as nodes in wireless sensor networks. As such, to understand how time efficient these algorithms are, several simulations were performed on different embedded boards. It should be noted that the goal is not to compare the algorithms against each other, since they all have the same (linear) computational complexities regarding the number of function evaluations. The goal here is to evaluate whether swarm-based algorithms, in general, are feasible in embedded devices and for use in constrained-time applications.

For this effort, we present the execution times of each swarm algorithm on different embedded devices averaged over 10,000 Monte Carlo runs, giving as a reference the execution times of the Grid Search algorithm with a 0.1 m spacing interval. The average computational times (in milliseconds) of the Grid Search algorithm are presented in Table 8 for the different boards and numbers of sensors. For the swarm algorithms, Table 9 shows the average execution times (also in milliseconds) that they take to reach 1000 function evaluations and the respective standard deviations. (One thousand function evaluations are a value sufficient for convergence, as shown by the previous sections. However, if desired, the estimation of the execution times for a different number of function evaluations is straightforward, since time is linearly proportional to the number of function evaluations).

The obtained results demonstrate that while the swarm algorithms have very similar performance to that of the Grid Search in terms of accuracy, they are very superior in terms of time efficiency, with computational times that are 100 times faster than that of the Grid Search. If the small grid space of 0.1 m allows the Grid Search to accurately locate the acoustic target, it demands a time-consuming computational burden, which is avoided in the swarm-based algorithms. Knowing the average time superiority of the swarm, it is also important to see if that superiority is constant or volatile considering the stochastic nature of the algorithms in their operations and the execution flow of their subroutines. The computational times’ standard deviations ($\sigma_{t}$) in Table 9 show that the obtained times are very constant, which also allows the application of these methods in systems where determinism and reliability are important issues. The execution times presented in Table 9 can be seen as a reference for the time that it takes to estimate the source location of an acoustic event in devices with processors ranging from 1.5 GHz (Rasp. Pi 4 B) to 0.7 GHz (Rasp. Pi B) clock rates. The processing time is not just dependent on the clock rates, but also on the device architecture itself, whereby the presented reference time may vary slightly for other devices, even with the same clock rates. Nonetheless, the obtained times show that the localization estimation can be performed in dozens of milliseconds, which can be considered as being on a real-time scale. Thus, after analyzing the accuracy of the methods in the previous sections and the execution time performance in this section, the following claim is demonstrated: Through swarm-based optimization with smart/intelligent initialization, acoustic source localization can be done at the edge on embedded devices with good accuracy and in real time.

## 6. Conclusions

The comprehensive study presented here extends and expands previous work on swarm optimization for energy-based acoustic source localization by applying some of the most popular and novel swarm-based algorithms to the EBAL problem. Three main goals guided the present work.

Considering the simulations performed, three algorithms, namely, MFO, SDO, and EEHO, showed great performance. While the former slightly overcame the other two in cost convergence, the average errors of the three methods were very similar. In addition, considering the features of the different algorithms tested and the obtained results, it was shown that when using smart/intelligent initialization, the algorithms that rely more on the local space perform better than the ones with stronger initial exploration phases.

The second goal was to see whether the intelligent initialization that was previously proposed and validated for the EHO method could also work for any swarm-based algorithm. Overall, the algorithms used in the simulations all had their average performance improved when using intelligent initialization. As such, it is now possible to claim that this initialization technique should always be considered when implementing any swarm-based algorithm for the EBAL problem.

After widely studying the accuracy of the swarm-based methods in solving the EBAL problem, it remained to analyze their computational time performance. To that end, the algorithms were implemented, and a large set of simulations were executed on five different boards that could be used in real edge computing scenarios. The obtained results demonstrated the value of the mathematical simplicity of swarm-based algorithms. As such, it is possible to locate acoustic sources in units of milliseconds or dozens of milliseconds, depending on the processors used or the number of sensors considered, allowing the use of the presented approach in *real-time* edge computing applications.

With the completion of these three goals, the present work is a crucial milestone in acoustic source localization through swarm intelligence, breaking barriers towards its real implementation in demanding edge computing scenarios. The typical physical architecture of these systems relies on a powerful centralized machine and complex algorithms to process the acoustic signals obtained at the edge of the architecture by acoustic sensors. The low computational complexity of the approach considered in this work allows for the localization to be calculated at the edge of the architecture itself, where a central processor would receive only the estimated location coordinates, which are what is required in most applications. The benefits of this are obvious, and, as proved by the present work, both accuracy and real-time performance can be guaranteed.

One of the major shortcomings of the presented methods is that they depend on a noise model, given that they are based on evaluating an objective function whose form is determined by the noise statistics. A possible direction for our future work will include the derivation of a new objective function that does not depend on noise statistics, but is a valid cost criterion for evaluating the quality of the particles. Another drawback of swarm-based methods is that they usually require a training phase to optimize some of the parameters used in their operation. Although the impact of these parameters is not crucial in terms of their functioning, they do have a tuning effect on their performance.

While the present work focused on a solution based on energy measurements, the same work can be applied or extended to any other range-based localization method. As future work, different research challenges exist, such as the development of noise mitigation techniques to improve the accuracy (e.g., by considering variables other than energy measures), the application of this approach to other range-based localization methods, or the integration and implementation of this solution on real edge computing localization systems.

## Figures and Tables

**Table 1:** Citation metrics of swarm-based algorithms.

	Year	Acronym	(*)	(**)	Method (Reference)
–1999	1995	PSO	61,839	2474	Particle Swarm Optimization [9]
	1996	ANT	14,356	598	Ant System [73]
2000–2009	2009	**CS **	5100	464	Cuckoo Search via Lévy flights [16]
2010–2014	2010	BAT	3753	375	Bat Algorithm [17]
	2011	TLBO	2227	247	Teaching–Learning-Based Optimization [62]
	2014	**GWO**	4112	685	Grey Wolf Optimizer [61]
2015–2020	2015	**MFO**	1167	233	Moth–Flame Optimization Algorithm [63]
	2016	**WOA**	2227	557	Whale Optimization Algorithm [64]
	2017	**SSA**	894	298	Salp Swarm Algorithm [65]
	2018	**TGA**	45	23	Tree Growth Algorithm [66]
		**COA**	85	43	Coyote Optimization Algorithm [67]
	2019	**SDO**	9	9	Supply–Demand-Based Optimization [68]
		**EEHO**	16	16	Enhanced Elephant Herding Optimization [69]
	2020	**MSA**	-	-	Momentum Search Algorithm [72]

(*) Number of citations in set/2020 [scholar.google.com]. (**) Number of citations per year in set/2020 [scholar.google.com].

**Table 2:** Computational architectures of the swarm-based algorithms’ implementation.

	Rasp. Pi 4 B	Rasp. Pi ZW	Rasp. Pi 3	Rasp. Pi 2	Rasp. Pi B
SOC	BCM2711	BCM2835	BCM2837	BCM2836	BCM2835
Core	Cortex-A72 [64-bit]	ARM1176JZF-S	Cortex-A53 [64-bit]	Cortex-A7	ARM1176JZF-S
Cores	4	1	4	4	1
Clock	1.5 GHz	1 GHz	1.2 GHz	900 MHz	700 MHz
RAM	4 GB	512 MB	1 GB	1 GB	512 MB

**Table 3:** TGA operators.

Target Agents	Operator	Goal
$x_{i}$ for $i=1,2,\ldots ,N_{1}$ (Best Trees)	(12)	Exploitation
$x_{i}$ for $i=N_{1}+1,N_{1}+2,\ldots ,N_{1}+N_{2}$ (Competition Trees)	(13)	Exploration, exploitation
$x_{i}$ for $i=N_{1,2}+1,N_{1,2}+2,\ldots ,N_{1,2}+N_{3}$, where $N_{1,2}=N_{1}+N_{2}$ (Remove Trees)	(14)	Exploration
$x_{i}$ for $i=N_{1,2,3}+1,N_{1,2,3}+2,\ldots ,N_{1,2,3}+N_{4}$, where $N_{1,2,3}=N_{1,2}+N_{3}$ (Reproduction Trees)	(15)	Exploration, exploitation

**Table 4:** Comparison of the algorithms’ properties.

Method	Sub-Groups	Random Variable Distributions	Exploitation/Exploration Balance over Iterations	Quality Evolution
**CS**	No **✗**	U, N, L	Constant	Greedy
**GWO**	No **✗**	U	Variable	Elitist
**EEHO**	Yes **✓**	U	Constant	Elitist
**MFO**	No **✗**	U	Variable	Elitist
**WOA**	No **✗**	U	Variable	Elitist
**SSA**	No **✗**	U	Variable	Elitist
**TGA**	No **✗**	U	Constant	Elitist
**COA**	Yes **✓**	U	Constant	Elitist
**SDO**	No **✗**	U	Variable	Greedy
**MSA**	No **✗**	U	Variable	Elitist

U—Uniform distribution; N—Normal distribution; L—Lévy distribution.

**Table 5:** Test parameters.

Search Space	50 m × 50 m
P	5
$g_{i}$	1
$\beta_{L}$	2
Noise Variance	$\sigma_{\nu }^{2}\in \left\{-80,-75,-70,-65,-60,-55\right\}$ (dB)
Number of Sensors	N∈{6,9,12,15}

**Table 6:** Algorithms’ parameters.

	Population Size ($P_{S}$)	No. of Groups ($G_{N}$)	Groups Size ($G_{S}$)	$\frac{\mathrm{Evaluations}}{\mathrm{Iteration}}$	Specific Parameters
**CS**	25	n.a.	n.a.	$2\times P_{S}$, i.e., 50	$P_{a}=0.25$
**GWO**	30	n.a.	n.a.	$P_{S}$, i.e., 30	n.a.
**EEHO**	120	(3,4,5,6)	(40,30,25,20)	$P_{S}$, i.e., 120	α=0.7 β=0.1 γ=0.015
**MFO**	30	n.a.	n.a.	$P_{S}$, i.e., 30	b=1
**WOA**	30	n.a.	n.a.	$P_{S}$, i.e., 30	b=1
**SSA**	30	n.a.	n.a.	$P_{S}$, i.e., 30	n.a.
**TGA**	100	n.a.	n.a.	$P_{S}+N_{4}$, i.e., 150	$N_{1}=N_{2}=20$ $N_{3}=60$ $N_{4}=50$ θ=1.2 λ=0.5
**COA**	100	20	5	$P_{S}+G_{N}$, i.e., 25	$P_{e}=0.005\times {G_{S}}^{2}$
**SDO**	50	n.a.	n.a.	$P_{S}$, i.e., 50	n.a.
**MSA**	60	n.a.	n.a.	$P_{S}$, i.e., 60	$U_{max}=2.5$

n.a.—not applicable.

**Table 7:** Mean error (in meters) of the Grid Search (0.1 m interval).

	$\sigma_{\nu }^{2}=-80$ dB	$\sigma_{\nu }^{2}=-75$ dB	$\sigma_{\nu }^{2}=-70$ dB	$\sigma_{\nu }^{2}=-65$ dB	$\sigma_{\nu }^{2}=-60$ dB	$\sigma_{\nu }^{2}=-55$ dB
N=6	0.083	0.134	0.222	0.394	0.798	2.844
N=9	0.056	0.076	0.130	0.204	0.402	1.980
N=12	0.051	0.078	0.100	0.161	0.328	0.533
N=15	0.048	0.062	0.086	0.140	0.215	0.476

**Table 8:** Average execution time (in milliseconds) of the Grid Search (0.1 interval).

	N=6	N=9	N=12	N=15
Rasp. Pi B	1999	2983	4032	5057
Rasp. Pi ZW	1347	2013	2719	3404
Rasp. Pi 2	946	1411	1895	2368
Rasp. Pi 3	589	880	1171	1477
Rasp. Pi 4 B	241	358	477	595

**Table 9:** Average execution time ($\bar{t}$) ± standard deviation ($\sigma_{t}$) to reach 1000 function evaluations (in milliseconds).

		N=6	N=9	N=12	N=15
Rasp. Pi B	CS	16.258±0.094	20.537±0.095	28.714±0.107	29.561±0.116
	GWO	12.282±0.051	16.731±0.057	20.913±0.059	29.264±0.073
	EEHO	10.223±0.122	14.251±0.150	18.541±0.187	22.764±0.406
	MFO	14.346±0.568	18.620±0.435	22.442±0.381	26.406±0.386
	WOA	10.894±0.051	15.459±0.063	19.696±0.068	28.098±0.087
	SSA	9.688±0.062	15.086±0.131	19.412±0.132	27.809±0.125
	TGA	10.888±0.295	15.082±0.400	21.949±0.589	24.133±0.773
	COA	11.500±0.098	15.760±0.128	20.159±0.169	24.464±0.187
	SDO	16.813±0.258	24.475±0.314	32.263±0.246	39.731±0.294
	MSA	10.613±0.350	14.702±0.685	18.793±0.586	23.076±0.956
Rasp. Pi ZW	CS	10.849±0.046	13.840±0.055	16.725±0.049	19.608±0.061
	GWO	8.270±0.027	11.462±0.059	14.457±0.078	20.220±0.087
	EEHO	6.930±0.188	9.615±0.079	12.630±0.491	15.300±0.159
	MFO	10.060±0.398	12.821±0.375	15.444±0.366	18.874±0.533
	WOA	7.502±0.027	11.678±0.038	15.155±0.062	20.633±0.044
	SSA	6.448±0.041	9.259±0.049	11.938±0.028	18.016±0.050
	TGA	7.541±0.082	10.352±0.098	13.322±0.248	16.062±0.120
	COA	7.505±0.081	10.378±0.101	13.524±0.127	16.488±0.141
	SDO	11.461±0.154	16.652±0.198	21.932±0.147	27.163±0.174
	MSA	6.845±0.106	9.541±0.168	12.599±0.313	15.362±0.562
Rasp. Pi 2	CS	7.875±0.020	9.752±0.020	11.697±0.019	13.612±0.028
	GWO	6.171±0.024	8.090±0.034	10.000±0.016	11.848±0.017
	EEHO	4.813±0.017	6.659±0.020	8.613±0.022	10.513±0.023
	MFO	6.568±0.163	8.478±0.086	10.351±0.049	12.262±0.038
	WOA	5.156±0.017	7.063±0.019	8.971±0.017	10.889±0.018
	SSA	4.538±0.022	6.446±0.015	8.278±0.010	10.189±0.014
	TGA	4.957±0.024	6.849±0.024	8.784±0.024	10.689±0.033
	COA	5.493±0.016	7.441±0.017	9.429±0.020	11.389±0.021
	SDO	8.454±0.027	12.117±0.061	15.890±0.025	19.603±0.028
	MSA	4.923±0.017	6.784±0.016	8.695±0.027	10.583±0.030
Rasp. Pi 3	CS	4.783±0.016	5.927±0.017	7.155±0.016	8.348±0.017
	GWO	3.748±0.011	4.944±0.011	6.163±0.011	7.405±0.012
	EEHO	2.986±0.016	4.137±0.020	5.367±0.022	6.594±0.027
	MFO	4.079±0.115	5.275±0.058	6.434±0.039	7.637±0.029
	WOA	3.167±0.014	4.379±0.013	5.569±0.013	6.787±0.014
	SSA	2.799±0.011	3.980±0.019	5.162±0.008	6.373±0.012
	TGA	3.068±0.016	4.252±0.016	5.475±0.017	6.668±0.019
	COA	3.359±0.013	4.628±0.016	5.841±0.029	7.059±0.020
	SDO	5.201±0.024	7.482±0.051	9.807±0.023	12.150±0.028
	MSA	3.023±0.015	4.200±0.016	5.403±0.019	6.595±0.022
Rasp. Pi 4 B	CS	2.284±0.016	2.782±0.020	3.277±0.030	3.771±0.025
	GWO	1.868±0.008	2.350±0.010	2.829±0.018	3.313±0.013
	EEHO	1.329±0.015	1.862±0.014	2.291±0.022	2.769±0.025
	MFO	2.067±0.104	2.557±0.065	3.033±0.038	3.511±0.037
	WOA	1.439±0.009	1.926±0.011	2.407±0.013	2.888±0.017
	SSA	1.231±0.011	1.698±0.013	2.160±0.016	2.628±0.016
	TGA	1.413±0.009	1.904±0.010	2.381±0.010	2.863±0.011
	COA	1.692±0.012	2.181±0.014	2.672±0.018	3.164±0.021
	SDO	2.173±0.040	3.091±0.047	4.033±0.056	4.964±0.072
	MSA	1.341±0.015	1.811±0.018	2.285±0.022	2.753±0.024

## Appendix A. Definitions and Notation

**Definition** **A1.**
*An agent, search agent, or individual is an entity with a position in the search space and an associated quality. Its position at iteration t represents a solution for the problem, and it will be denoted by a D-dimensional vector $x^{t}$, where D is the number of dimensions of the problem. Its quality can be obtained by applying the cost function f on its position: $f(x^{t})$.*

**Notation** **A1.**
*Bold symbols should be interpreted as vectors.*

**Notation** **A2.**
*R∼U(a,b) means that R is a vector of random values $R_{i}$ drawn from a uniform distribution between a and b, such that $R_{i}∼U\left(a,b\right)$, for i=1,2,…,D, where D is the dimension of the vector. If R∼U(a,b), where a and b are both vectors, then it should be considered that each value $R_{i}$ follows a uniform distribution between $a_{i}$ and $b_{i}$, such that $R_{i}∼U\left(a_{i},b_{i}\right)$, for i=1,2,…,D. This also applies to the distributions described below.*

**Notation** **A3.**
*$R∼N(\mu ,\sigma^{2})$ means that R is a vector of random values drawn from a normal distribution with mean μ and variance $\sigma^{2}$.*

**Notation** **A4.**
*R∼L(α,γ) means that R is a vector of random values drawn from a Lévy symmetric alpha-stable distribution, with α as the index of stability and γ as a scale factor (the symmetric alpha-stable distribution is a specific case of alpha-stable distribution, where the skewness parameter β=0 and the location parameter δ=0).*

**Notation** **A5.**
*lb and ub represent the upper- and lower-bound vectors of the search space, respectively, so that any valid $x^{t}$ must satisfy $\mathrm{lb}\leq x^{t}\leq \mathrm{ub}$.*

## Appendix B. Swarm Algorithm Listing

**Table A1:** Swarm algorithm listing.

	Year ${}^{1}$	Acronym	(*)	(**)	Method (Reference)
–1999	1995	PSO	61,839	2474	Particle Swarm Optimization [9]
	1996	ANT	14,356	598	Ant System [73]
2000–2010	2001	HS	5315	280	Harmony Search [11]
	2002	BF	3214	179	Bacterial Foraging [12]
	2004	HB	323	20	Honey Bees [13]
	2006	SOA	152	11	Seeker Optimization Algorithm [92]
		GSO	211	15	Glowworm Swarm Optimization [93]
		HBMO	361	26	Honey Bee Mating Optimization [94]
		CSO	502	36	Cat Swarm Optimization [95]
		BA	1339	96	Bee Algorithm [14]
	2007	MS	228	18	Monkey Search [96]
		IWD	325	25	Intelligent Water Drops [97]
		ICA	2228	171	Imperialist Competitive Algorithm [98]
		ABC	5045	388	Artificial Bee Colony [15]
	2008	BBO	2821	235	Biogeography-Based Optimization [99]
	2009	DS	38	3	Dialectic Search [100]
		GSO	681	62	Group Search Optimizer [101]
		GSA	4354	396	Gravitational Search Algorithm [102]
		CS	5100	464	Cuckoo Search [16]
2010–2015	2010	SO	112	11	Spiral Optimization [103]
		FWA	729	73	Fireworks Algorithm for Optimization [104]
		CSS	900	90	Charged System Search [105]
		FA	3112	311	Firefly Algorithm [106]
		BAT	3753	375	Bat Algorithm [17]
	2011	GSA	160	18	Galaxy-Based Search Algorithm [107]
		DS	368	41	Differential Search [108]
		BSO	414	46	Brain Storm Optimization Algorithm [109]
		FOA	1126	125	Fruit Fly Optimization Algorithm [110]
		TLBO	2227	247	Teaching–Learning-Based Optimization [62]
	2012	ACROA	66	8	Artificial Chemical Reaction Optimization Algorithm [111]
		ACS	105	13	Artificial Cooperative Search [112]
		MBO	198	25	Migrating Bird Optimization [113]
		RO	396	50	Ray Optimization [114]
		MBA	412	52	Mine Blast Algorithm [115]
		BH	622	78	Black Hole [116]
		KH	1214	152	Krill Herd [117]
	2013	LCA	132	19	League Championship Algorithm [118]
		DE	293	42	Dolphin Echolocation [119]
		SSO	338	48	Social Spider Optimization [120]
	2014	OIO	97	16	Optics-Inspired Optimization [121]
		VS	175	29	Vortex Search [122]
		ISA	241	40	Interior Search Algorithm [123]
		SFS	255	43	Stochastic Fractal Search [124]
		SMO	266	44	Spider Monkey Optimization [125]
		PIO	274	46	Pigeon-Inspired Optimization [126]
		WWO	285	48	Water Wave Optimization [127]
		CSO	314	52	Chicken Swarm Optimization [128]
		CBO	388	65	Colliding Bodies Optimization [129]
		SOS	713	119	Symbiotic Organism Search [130]
		GWO	4112	685	Grey Wolf Optimizer [61]
2015–2020	2015	VOA	34	7	Virus Optimization Algorithm [131]
		WSA	40	8	Weighted Superposition Attraction [132]
		MBO	163	33	Monarch Butterfly Optimization [133]
		LSA	171	34	Lightning Search Algorithm [134]
		EHO	227	45	Elephant Herding Optimization [51]
		DA	877	175	Dragonfly Algorithm [135]
		SCA	1016	203	Sine–Cosine Algorithm [136]
		ALO	1161	232	The Ant Lion Optimizer [137]
		MFO	1167	233	Moth–Flame Optimization [63]
	2016	SWA	53	13	Sperm Whale Algorithm [138]
		MS	192	48	Moth Search [139]
		CSA	689	172	Crow Search Algorithm [140]
		WOA	2227	557	Whale Optimization Algorithm [64]
	2017	KA	58	19	Kidney-Inspired Algorithm [141]
		SHO	65	22	Selfish Herd Optimizer [142]
		TEO	126	42	Thermal Exchange Optimization [143]
		SHO	166	55	Spotted Hyena Optimizer [144]
		GOA	700	233	Grasshopper Optimization Algorithm [145]
		SSA	894	298	Salp Swarm Algorithm [65]
	2018	TGA	45	15	Tree Growth Algorithm [66]
		FF	59	20	Farmland Fertility [146]
		COA	85	28	Coyote Optimization Algorithm [67]
		BOA	172	57	Butterfly Optimization Algorithm [147]
		EWA	174	58	Earthworm Optimization Algorithm [148]
	2019	NRO	6	6	Nuclear Reaction Optimization [149]
		SDO	9	9	Supply–Demand-Based Optimization [68]
		PRO	12	12	Poor and Rich Optimization Algorithm [150]
		EEHO	16	16	Enhanced Elephant Herding Optimization [69]
		FDO	20	20	Fitness Dependent Optimizer [151]
		BWO	20	20	Black Widow Optimization Algorithm [152]
		PFA	32	32	Pathfinder Algorithm [153]
		EO	56	56	Equilibrium Optimizer [154]
		SOA	60	60	Seagull Optimization Algorithm [155]
		SSA	150	150	Squirrel Search Algorithm [65]
		HHO	323	323	Harris Hawks Optimization [71]
	2020	BOA	1	1	Billiards-Inspired Optimization Algorithm [156]
		WSA	5	1	Water Strider Algorithm [157]
		DGCO	7	7	Dynamic Group-Based Cooperative Optimization [158]
		TSA	12	12	Tunicate Swarm Algorithm [159]
		MPA	38	38	Marine Predators Algorithm [160]
		WFS	-	-	Wingsuit Flying Search [161]
		AOA	-	-	Archimedes Optimization Algorithm [162]
		MSA	-	-	Momentum Search Algorithm [72]

^1^ Year of online publication (may be different from issue year). (*) Number of citations in set/2020 [scholar.google.com]. (**) Number of citations per year in set/2020 [scholar.google.com].
